# Short-term and medium-term clinical outcomes of multisystem inflammatory syndrome in children: a prospective observational cohort study

**DOI:** 10.1186/s13052-023-01569-7

**Published:** 2024-01-04

**Authors:** Anastasia Glazyrina, Elena Zholobova, Ekaterina Iakovleva, Polina Bobkova, Ekaterina Krasnaya, Karina Kovygina, Olga Romanova, Oleg Blyuss, Konstantin Tutelman, Polina Petrova, Anastasiia Bairashevskaia, Mikhail Rumyantsev, Anatoliy A. Korsunskiy, Elena Kondrikova, Anzhelika Nargizyan, Valeriya Yusupova, Evgeniya Korobyants, Anna Sologub, Seda Kurbanova, Aleksandr Suvorov, Louise Sigfrid, Danilo Buonsenso, Diego G. Peroni, Andrew James McArdle, Pasquale Comberiati, Daniel Munblit

**Affiliations:** 1 Morozovskaya Children’s Municipal Clinical Hospital of the Moscow City Health Department, Moscow, Russia; 2 Department of Children Diseases, Institute of Child’s Health, Sechenov First Moscow State Medical University (Sechenov University), Moscow, Russia; 3 Department of Paediatrics and Paediatric Infectious Diseases, Institute of Child’s Health, Sechenov First Moscow State Medical University (Sechenov University), Moscow, Russia; 4 Centre for Prevention, Detection and Diagnosis, Queen Mary University of London, London, UK; 5 Veltischev Clinical Pediatric Research Institute of Pirogov Russian National Research Medical University, Moscow, Russia; 6 World-Class Research Center “Digital biodesign and personalized healthcare”, Sechenov First Moscow State Medical University (Sechenov University), Moscow, Russia; 7 ISARIC Global Support Centre, Nuffield Department of Medicine, University of Oxford, Oxford, UK; 8 Department of Woman and Child Health and Public Health, Fondazione Policlinico Universitario A. Gemelli IRCCS, Rome, Italy; 9 Dipartimento di Scienze Biotecnologiche di Base, Cliniche Intensivologiche e Perioperatorie, Università Cattolica del Sacro Cuore, Rome, Italy; 10 Global Health Research Institute, Istituto di Igiene, Università Cattolica del Sacro Cuore, Rome, Italy; 11 Department of Clinical and Experimental Medicine, Section of Pediatrics, University of Pisa, Pisa, Italy; 12 Department of Infectious Disease, Section of Pediatric Infectious Disease, Imperial College London, London, UK; 13 Care for Long Term Conditions Division, Florence Nightingale Faculty of Nursing, Midwifery and Palliative Care, King’s College London, London, UK; 14 Research and Clinical Center for Neuropsychiatry, Moscow, Russia

**Keywords:** Children, COVID-19, Multisystem inflammatory syndrome in children, MIS-C, Paediatric inflammatory multisystem syndrome temporally associated with COVID-19, PIMS-TS, SARS-CoV2, Sequelae

## Abstract

**Background:**

Even though the incidence of Multisystem Inflammatory Syndrome in children (MIS-C) is decreasing cases are still reported across the world. Studying the consequences of MIS-C enhances our understanding of the disease’s prognosis. The objective of this study was to assess short- and medium-term clinical outcomes of MIS-C.

**Methods:**

Prospective observational cohort study at Municipal Children’s Hospital Morozovskaya, Moscow, Russia. All children meeting the Royal College of Paediatrics and Child Health (RCPCH), Centers for Disease Control and Prevention (CDC), or the World Health Organization (WHO) MIS-C case definition admitted to the hospital between 17 May and 26 October 2020 were included in the study. All survivors were invited to attend a clinic at 2 and 6 weeks after hospital discharge.

**Results:**

37 children median age 6 years (interquartile range [IQR] 3.3–9.4), 59.5% (22/37) boys were included in the study. 48.6% (18/37) of patients required ICU care. One child died. All children had increased levels of systemic inflammatory markers during the acute event. Echocardiographic investigations identified abnormal findings in 35.1% (13/37) of children. 5.6% (2/36) of children were presenting with any symptoms six weeks after discharge. By six weeks the inflammatory markers were within the reference norms in all children. The echocardiographic evaluation showed persistent coronary dilatation in one child.

**Conclusions:**

Despite the severity of their acute MIS-C, the majority of children in our cohort fully recovered with none having elevated laboratory markers of inflammation at 6 weeks, few (< 10%) reporting persistent symptoms at 6 weeks, and only one with persistent echocardiographic abnormalities.

**Supplementary Information:**

The online version contains supplementary material available at 10.1186/s13052-023-01569-7.

## Introduction

The severe acute respiratory syndrome coronavirus 2 (SARS-CoV-2) global pandemic has had detrimental consequences to public health. Data from epidemiological and cohort studies suggest that children experience a milder acute phase of novel coronavirus disease 2019 (COVID-19) than adults [1, 2] and develop long-term consequences following SARS-CoV-2 less often [3].

In May 2020, a case series in London described children presenting with hyperinflammatory shock, which was described later in additional countries around the world [4–6] and defined as a Multisystem Inflammatory Syndrome in children (MIS-C) or paediatric inflammatory multisystem syndrome temporally associated with COVID-19 (PIMS-TS).

It is considered that MIS-C develops several weeks after the acute phase of COVID-19 [7, 8]. Recently published data from the United States describing clinical characteristics as well as geographic and temporal distribution of patients with MIS-C showed that MIS-C occurred 2–5 weeks after SARS-CoV-2 infection [8]. Estimated incidence of MIS-C in 2020–2021 varied among the studies with some data suggesting that as many as 316 persons per 1,000,000 SARS-CoV-2 infections in persons younger than 21 years may develop MIS-C [9] and up to 1 per 4000 children with SARS-CoV-2 [10]. A strong geographical and temporal association between SARS-CoV-2 infection rates and MIS-C cases was also described by Flood et al. [11].

Recent data indicates a declining incidence of MIS-C. During the pre-Delta period, the MIS-C rate was at 13 cases per 10,000 notified SARS-CoV-2 infections among those aged 0–19 years. This rate dropped to 5 per 10,000 during the Delta variant wave and further decreased to 0.8 per 10,000 in the wave of Omicron [12]. Several factors could explain this decline, such as changes in the dominant virus strains or widespread vaccination campaigns targeting children [13]. The severity of MIS-C has also been on a downward trend [14]. However, despite the reduced risk of MIS-C complications over time, there are still reports of admissions to intensive care units (ICU) [15].

Data on MIS-C consequences is growing. A study from Turkey [16] investigated the short-term sequelae within 2 weeks following discharge, while the data from India [17] were collected 3–4 months post-discharge in a small group of patients. A recent UK study showed that organ-specific consequences were found 6 months after the acute event in a small proportion of children post-SARS-CoV-2 infection [18], but data on MIS-C sequelae is still need to be studied in different populations [19–22].

This study aimed to describe short- and medium-term clinical outcomes of children admitted with MIS-C to a tertiary paediatric hospital in Moscow, Russia, and investigate the repercussions of this condition.

## Materials and methods

### Study design and ethics

This prospective observational cohort study took place at Municipal Children’s Hospital Morozovskaya, Moscow, Russian Federation. All patients ≤18 years of age admitted to the hospital with MIS-C between 17 May and 26 October 2020 were included in the study.

We enrolled all patients who met the current MIS-C case definition by CDC or WHO, or RCPCH clinical diagnostic criteria (Supplementary, Appendix Table A1) with evidence of positive Polymerase chain reaction (PCR) test results and/or IgM/IgG antibodies against SARS-CoV-2 within 4 weeks prior to the MIS-C acute event or during hospitalisation, as a proof of previous SARS-CoV-2 exposure. All survivors were offered two in-hospital follow-up visits at the Rheumatology Department of Municipal Children’s Hospital Morozovskaya at 2 and 6 weeks after discharge.

Patients with Kawasaki disease, toxic shock syndrome, sepsis, macrophage activation syndrome, and the other causes of inflammation were excluded from the study. Diagnosis was made by the clinical experts from the Rheumatology department.

**Fig. 1:**
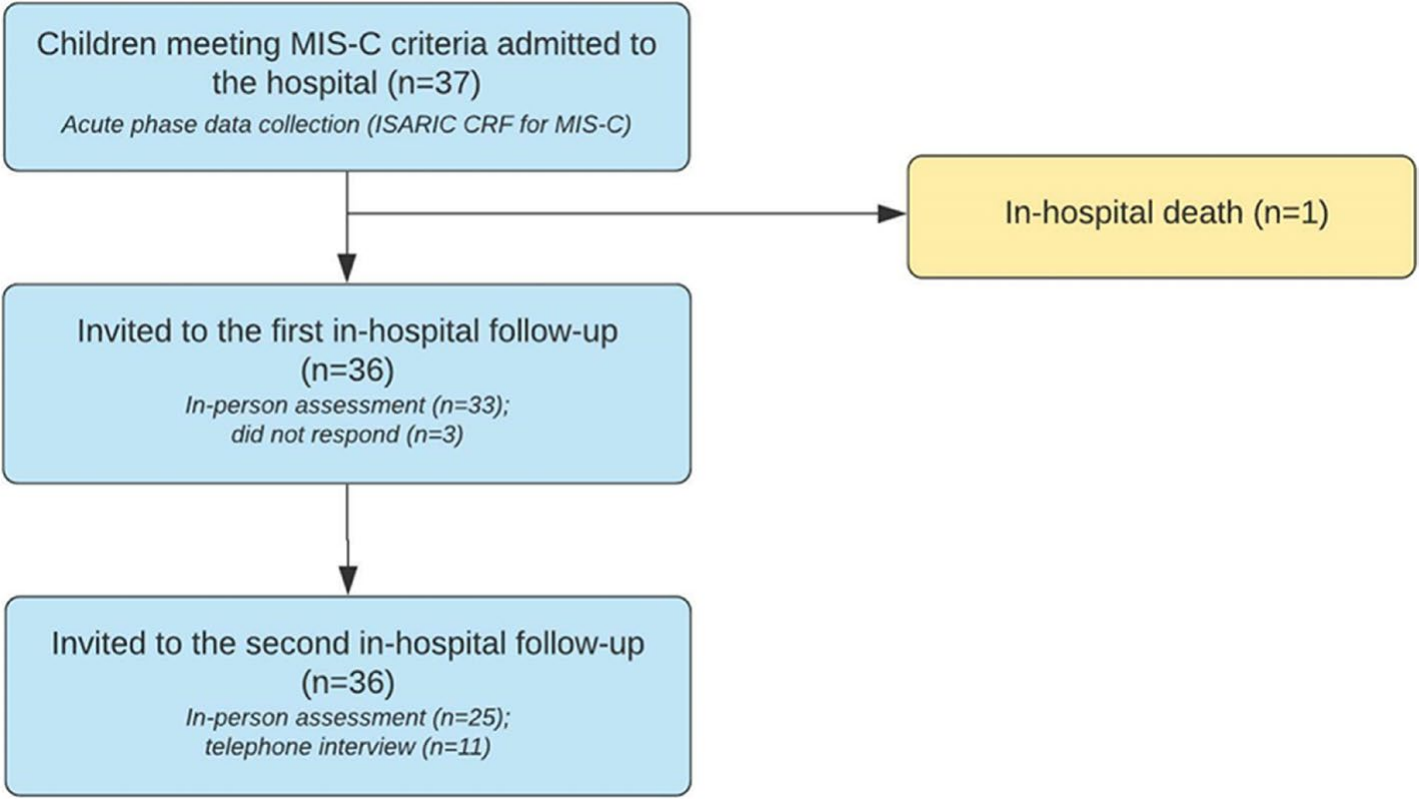
Study flow chart, showing patients with MIS-C admitted to Municipal Children’s Hospital Morozovskaya between 17 May and 26 October 2020, recruited into the study and subsequent follow-ups

This study was approved by the Moscow City Independent Ethics Committee (abb. 1, protocol number 74).

### Data collection process

The data were collected from electronic medical records and hospital notes and included demographics, signs and symptoms during the acute phase of MIS-C and at the follow-up assessment, comorbidities, medical history, computed tomography (CT) imaging, electrocardiography (ECG), echocardiography (ECHO), laboratory and pathogen test results, supportive care, treatment modalities and outcome for all children that met the inclusion criteria.

Data were extracted by a team of medical students with previous experience in data extraction using the case report form (CRF) developed by the International Severe Acute Respiratory and Emerging Infection Consortium (ISARIC) and the World Health Organization (WHO) from other COVID-19, post-COVID-19 condition, and MIS-C studies [3, 23–26]. The appointment for the follow-up visits was set by the hospital staff. Parents were informed of the clinical procedures, laboratory testing and examinations to be undertaken during the follow-up hospital visit. If children failed to attend in-hospital follow-up visits, parents were approached via a telephone assessment. Non-responders were contacted by telephone three times before being considered lost to follow-up.

Baseline characteristics and hospital follow-up data were collected using the modified ‘Case Record Form for suspected cases of MIS-C in children and adolescents temporally related to COVID-19’ developed by ISARIC and WHO and translated into Russian [27]. REDCap (Research Electronic Data Capture, Vanderbilt University, US, hosted at Sechenov University) was used for data collection, storage and management.

### Outcomes and definitions

The primary outcome of this study was the presence of symptoms 6 weeks after hospital discharge. Secondary outcomes included dynamics of blood test results and ECHO changes.

For this study, patients were defined as MIS-C if any of the following definitions were met: (a) RCPCH, (b) CDC, or (c) the WHO definitions (Supplementary, Appendix Table A1).

A fever was defined as a body temperature of 38 °C or higher.

Blood test abnormalities were defined as results higher or lower than the age-appropriate reference range measurement throughout the hospital stay, in accordance with the ISARIC/WHO CRF [27].

A detailed definition of ECHO changes is outlined in Supplementary, Appendix Box A1 [28, 29].

### Statistical analysis

Statistical analysis was performed in R v.3.5.1, graphical images were made in Python using matplotlib [30]. Statistical significance was set as α = 0.05.

Descriptive statistics were calculated for baseline characteristics. Continuous data was summarised as medians and interquartile ranges, nominal data as absolute counts and percentages – n (%). Where any data was missing, valid counts were shown as n/N (%).

Radial map plots were used to present dynamics in symptom presence during the acute phase and at follow-ups. Dynamic changes for serum markers were shown as parallel plots.

## Results

A total of 37 children meeting MIS-C criteria were admitted to the hospital between 17 May and 26 October 2020 (Fig. 1). Out of all admitted individuals, one child died during the admission, three did not attend the first follow-up but managed to visit the hospital for the second follow-up.

Table 1 summarizes patients’ demographic and clinical features. The median age at admission was 6 years (interquartile range [IQR] 3.3–9.4, range 8 months – 17 years), 59.5% (22/37) were boys. The median time from hospital admission to discharge or death was 13 (11.8–17.3) days. Comorbidities were reported in 40.5% (15/37) of children, the most common being allergic diseases (18.9%), chronic neurological disorders (5.4%), chronic kidney diseases (5.4%) and gastrointestinal conditions (5.4%).

**Table 1:** Demographic characteristics of patients admitted to Municipal Children’s Hospital Morozovskaya

Characteristic		Total
Age (median, IQR)		6 (3.3–9.9)
Age groups (years)	Age < 1 year	3 (8.1%)
	Age:1–4	14 (37.8%)
	Age: 5–9	10 (27%)
	Age:10–14	8 (21.6%)
	Age:15–18	2 (5.4%)
Sex	Female	15 (40.5%)
Duration of hospitalisation, days	Median (IQR)	13 (12–17)
Time since discharge to the first day of follow-up 1, days	Median (IQR)	15 (14–18)
Time since discharge to the first day of follow-up 2, days	Median (IQR)	47 (41–52)
In-hospital deaths		1 (2.7%)
**Comorbidities**		
Allergic diseases		7 (18.9%)
Psoriasis		1 (2.7%)
Сhronic neurological disorders		2 (5.4%)
Сhronic kidney diseases		2 (5.4%)
Heart diseases		1 (2.7%)
Gastrointestinal conditions		2 (5.4%)

At the time of the hospital admission, 97.3% (36/37) of children had IgG and 2.7% (1/37) had IgM antibodies against SARS-CoV-2. 10.8% (4/37) of children had positive PCR test results within the previous 4 weeks prior to current illness onset, and 32.4% (12/37) had evidence of close contact with an individual with COVID-19 within the same time frame. None of the children had a positive PCR at the time of the admission.

**Fig. 2:**
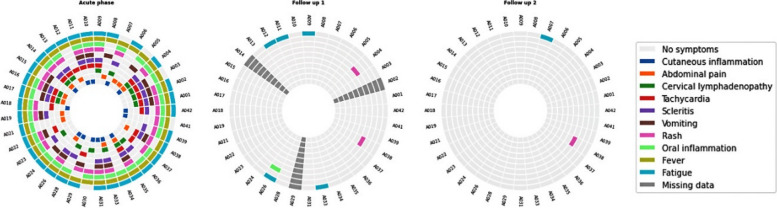
Radial plots represent the coexistence of the 10 most common symptoms during the acute MIS-C phase and at ‘Follow-up 1’ (2 weeks after discharge) and ‘Follow-up 2’ (6 weeks after discharge). The symptoms are shown for each patient; each segment represents a single patient

All patients had a fever (≥38 °C) at the time of hospital admission for MIS-C. Other common symptoms included fatigue/malaise 86.5% (32/37), oral inflammation 83.8% (31/37), skin rash 70.3% (26/37) and scleritis 62.2% (23/37). There was a substantial overlap between the top 10 most common symptoms, with 16 (43.2%) of 37 patients having 5 or more symptoms at the time of admission (Fig. 2).

All children were treated with parenteral antibiotics. Other treatments included anticoagulants 91.9% (34/37), systemic corticosteroids 89.2% (33/37) and intravenous fluids 89.2% (33/37), intravenous immunoglobulins 86.5% (32/37), and inotropes/vasopressors 37.8% (14/37). 21.6% (8/37) of patients required a blood transfusion.

Almost half (48.6%; 18/37) of children required ICU care, 27% (10/37) received oxygen supplementation and 8.1% (3/37) invasive ventilation. Two of the patients on invasive mechanical ventilation also underwent dialysis. Details of treatment used during hospital stay are presented in Supplementary, Appendix Table A2.

Laboratory results showed elevated inflammatory markers in all children during the hospital stay, with C-reactive protein (CRP) being elevated in 100% (37/37), D-dimer in 94.6% (35/37), erythrocyte sedimentation rate (ESR) in 89.2% (33/37), ferritin in 83.8% (31/37), and lactate dehydrogenase (LDH) in 48.6% (18/37) (Supplementary, Appendix Table A3). All laboratory parameters data are presented in Supplementary, Appendix Table A4.

The majority of children had evidence of a prothrombotic state, with elevated fibrinogen found in 75.7% (28/37), prolongation of international normalised ratio (INR) in 62.2% (23/37), and prolongation of activated partial thromboplastin time (aPTT) in 45.9% (17/37).

Full blood count showed neutrophilia in 94.6% (35/37), anemia in 89.2% (33/37), thrombocytopenia in 78.4% (29/37) and lymphocytopenia in 62.2% (23/37).

**Fig. 3:**
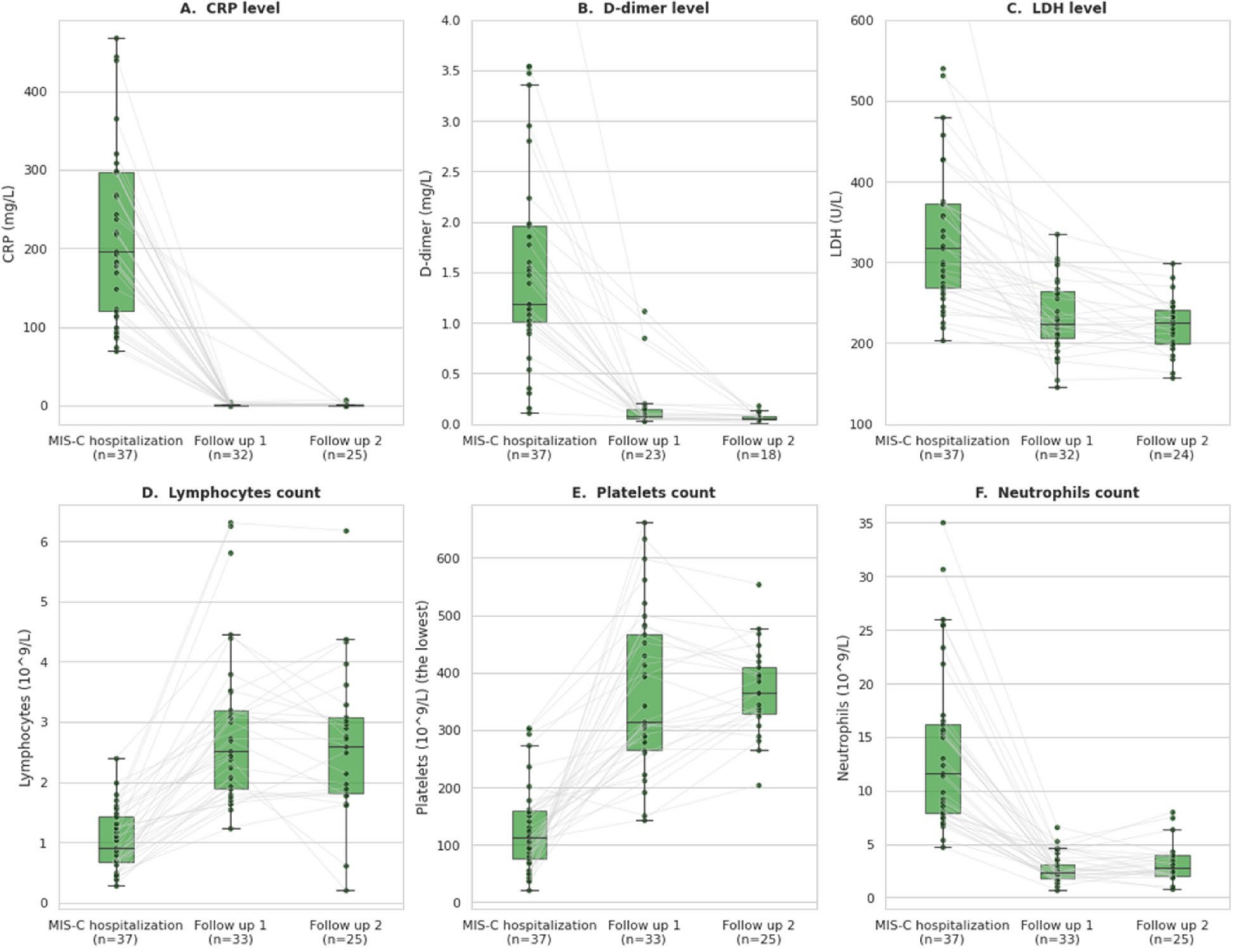
Box plots and parallel plots representing changes in serum markers over time: during the acute MIS-C phase and at ‘Follow-up 1’ (2 weeks after discharge) and ‘Follow-up 2’ (6 weeks after discharge). The horizontal lines in the boxes indicate medians; lower and upper edges of boxes indicate the interquartile range, and the bars extend to the highest and lowest value within 1.5 times the interquartile ranges

We found hypoalbuminemia in 91.9% (34/37), elevated alanine aminotransferase (ALT) in 75.5% (28/37) and raised urea in 43.2% (16/37) during the hospital stay (Fig. 3).

Signs of cardiac involvement included elevated troponin, which was present in 74.2% (23/31) of children. ECHO investigations identified abnormal findings in 35.1% (13/37) of children, with 16.2% (6/37) having evidence of myocardial dysfunction, 13.5% (5/37) features of pericarditis, and 5.4% (2/37) coronary artery abnormalities. No children had aneurysms or features of valvulitis. All diagnostic findings are presented in Supplementary, Appendix Table A5.

Out of 36 survivors, 92% (33/36) attended the first in-hospital follow-up visit. We could not contact the parents of three children for the first follow-up, but their condition was assessed at the second follow-up. The median duration from the hospital discharge to the first follow-up was 15 (14–18) days.

21.1% (7/33) of children were still symptomatic at the time of the first follow-up, of which 9.1% (3/33) presented with one symptom and 12% (4/33) reported two symptoms (Fig. 2, Supplementary, Appendix Table A6). Persistent symptoms included fatigue in 15.2% (5/33), rash in 6.1% (2/33), and oropharyngeal mucosa inflammation, tachypnoea, headache, and joint pain in 3.0% (1/33) of the children.

Among laboratory markers of systemic inflammation, CRP and D-dimer and LDH normalized in more than 80% of children at the time of the first follow-up (2 weeks after discharge), while ESR and ferritin were still elevated in 45.2% (14/31) and 22.2% (6/27), respectively although the levels were substantially lower when compared with the acute phase. Coagulation profile parameters were within the reference range for INR in all children. Only 2 of 27 (7.4%) children had elevated fibrinogen levels and persistent prolongation of aPTT. Lymphocytes were normal in all patients, whereas thrombocytosis persisted in 45.5% (15/33) children, thrombocytopenia in 6.1% (2/33) and anemia in 18.2% (6/33).

There was still evidence of hepatic involvement (raised ALT) in 12.9% (4/31). Available troponin concentrations (15/37) were normal in 80% of patients. The follow-up ECHO assessment was normal in all but 2 of 32 (6%) children, who showed enlarged coronaries. In one of these two children, the coronary abnormality was absent during the acute MIS-C phase.

Sixty-nine percent (25/36) of survivors attended the second in-hospital follow-up visit (6 weeks after discharge), and 31% (11/36) were assessed via telephone. The median duration from the hospital discharge to the second follow-up was 47 (41–52) days.

At the second follow-up (6 weeks after discharge), only 5.6% (2/36) of children reported any symptoms, with one child having a skin rash and another told of fatigue. Symptoms requiring medical examination, including, but not limited to oral cavity inflammation, scleritis, tachycardia, cervical lymphadenopathy, tachypnoea, prolonged capillary refill time, decrease in diuresis, wheeze, were assessed objectively only in those who managed to attend hospital for the second follow-up visit. The inflammatory markers, such as CRP, D-dimer and LDH were within the reference norms in more than 95% of children, whereas ESR and ferritin were above the reference range in 16.7% (4/24) and 9.1% (2/22), respectively. Coagulation markers INR and fibrinogen normalized in all children, whereas prolongation of aPTT persisted in 5.3% (1/19). Thrombocytosis persisted in 80% (20/25) of children, whereas none had thrombocytopenia. 12% (3/25) still had anemia. Albumin, urea, ALT and troponin levels were within the reference range in all children. The ECHO evaluation showed persistent coronary dilatation in one child (5.3%; 1/19) who had evidence of this abnormality at the first follow-up assessment (2 weeks after discharge), but not during the acute MIS-C phase. The dynamics of blood markers over time are shown in Fig. 3 and Supplementary, Appendix Fig. A1.

## Discussion

Although knowledge regarding MIS-C is expanding, data on MIS-C sequelae are still need to be studied. This is one of the first studies investigating MIS-C short-term and medium-term consequences using ISARIC/WHO CRF. In our cohort, all children had features of multisystem involvement at the time of hospital admission and almost a half of them required ICU care. Despite MIS-C severity, significant improvements were observed rapidly, and only one in five children had any remaining sequelae 2 weeks after hospital discharge, while fewer than one in 10 children had any sequelae 1.5 months after the acute event.

Despite interest in the consequences of MIS-C, there are not enough data coming from some geographical locations. Major initiatives, such as The National Heart, Lung, and Blood Institute (NHLBI) Study on long-term outcomes after the MIS-C (MUSIC) were launched [31] which may provide solid evidence in the near future.

There are still cases of children being admitted to the ICU due to severe course of the disease. The features of MIS-C are getting often similar to the features of Kawasaki disease during the most recent variant periods of SARS-CoV-2 infection [15]. Knowledge about the course of MIS-C helps to distinguish it from Kawasaki disease and decide on the most appropriate management strategy.

All children in our cohort met CDC, WHO/ISARIC, or RCPCH criteria for MIS-C and had laboratory confirmation of previous SARS-CoV-2 infection. The clinical features of MIS-C patients during the acute phase included fever, anaemia, elevation of D-dimer, ESR and ALT, and abnormal levels of ferritin and neutrophils, which is in agreement with the outcomes of similar cohorts from other geographical regions [2, 6, 32].

Our cohort is largely comparable to some others previously described in the literature. Almost half of our patients required admission to the ICU, reflecting the severity of MIS-C. This is slightly lower than in a cohort from the United States, where 80% of patients were admitted to the ICU [5], but higher compared to a recent Turkish study where around a fifth of all patients required ICU admission [32].

The dynamics of recovery following the acute event in our cohort were similar to recently published data from the UK, which assessed sequelae 6 weeks and 6 months after MIS-C. Both studies demonstrated a reduction of symptoms and significant improvement in most children [18].

Our data are also comparable with results from a recent Swedish study demonstrating full recovery among most patients 8 weeks post-MIS-C diagnosis [22]. The similarity of MIS-C illness course during the acute phase in patients from different countries may allow for some extrapolation of the study results.

We found that the normalisation of many laboratory parameters occurred very rapidly following the acute event. Elevated levels of CRP, D-dimer and neutrophils, as well as hypoalbuminemia were found in most of the included children during the acute phase. However, 2 weeks following hospital discharge improvements were observed. Similar improvements in the level of inflammatory markers were shown in the study from Turkey which also assessed children 2 weeks after discharge [16] and in a study from India with a follow-up at 3–4 months [17]. Most hospital discharge laboratory parameters normalised in all the children after 6 weeks.

ECHO investigations during the acute phase identified abnormal findings in more than a quarter of children, including features of pericarditis, myocardial dysfunction and coronary abnormalities. A similar incidence was previously reported in the UK cohort [18]. Despite rapid regression of symptoms and normalisation of most laboratory parameters, follow-up ECHO assessment identified coronary abnormalities in two children, comparable to the results from the UK. Data on the potential long-term impact of MIS-C on coronary arteries is still very limited. Although the results of our and other studies are reassuring, further observation at a longer follow-up period is required.

This study is one of a few research efforts evaluating short- and medium-term consequences of MIS-C. The main strength of our study is its prospective nature alongside harmonised and validated data collection, using the ISARIC/WHO MIS-C CRF. The follow-up data was collected during in-depth in-person assessments in the hospital by physicians to provide robust, objective data. Some of the children’s parents who were unable to attend follow-up visit were interviewed by phone using MIS-C CRF.

This study has several limitations. First, the data were collected in a single centre, and there are no data on the features of the course and consequences of MIS-C in children admitted to other hospitals. However, Municipal Children’s Hospital Morozovskaya is the primary hospital for admission of children with MIS-C meaning that the majority of children with a suspected MIS-C diagnosis residing in Moscow were hospitalised in this center. Second, some data were collected retrospectively from electronic medical records, which may result in incomplete information on some patients. Third, there is a risk of attrition bias as not all patients were able to attend clinical facilities in person. Information about the signs requiring medical assessment (such as tachypnoea, prolonged capillary refill time, etc) was not available in these patients. Nonetheless, the lack of symptoms that alarmed parents during an exhaustive telephone interview suggested that these children had clinically recovered. Fourth, the data has not been collected on children 18 to 21 years although this age range is included in the CDC MIS-C case definition and children 18–19 years old are included in the WHO MIS-C case definition.

## Conclusions

Although MIS-C is a severe, life-threatening condition the majority of the children, in our cohort recovered quickly with nearly all having no symptoms accompanied by normal laboratory and ECHO findings. This adds to the reassurance from other published studies. However, coronary abnormalities were seen in a small number of children, and long-term monitoring should be considered. Further research is required to assess long-term MIS-C in large prospective multinational studies to include children from different demographics.

### Supplementary Information

**Additional file 1: Supplementary, Appendix:** **Table A1. **MIS-C/PIMS criteria; **Table A2.** Number of children receiving different treatment options during hospitalisation; **Table A3.** The number of children with abnormally high and low laboratory test results revealed during the hospital stay; **Table A4.** Laboratory parameters across timepoints. Acute phase refers to the worst laboratory results during hospitalisation; **Table A5.** Findings on chest CT and echocardiography; **Table A6.** Frequency of symptoms across timepoints; **Box A1.** Definitions of echocardiographic changes; **Fig. A1.** Box plots and parallel plots representing changes in serum markers over time: during the acute MIS-C phase and at ‘Follow-up 1’ (2 weeks after discharge) and ‘Follow-up 2’ (6 weeks after discharge).

## Data Availability

Deidentified data may be made available on reasonable request to the corresponding author. All data access will be subject to a data use agreement and approval by the Municipal Children’s Hospital Morozovskaya Review Board.

## Abbreviations

aPTT: activated partial thromboplastin time
CT: computed tomography
CRP: c-reactive protein
COVID-19: coronavirus disease 2019
ECHO: echocardiography (ECHO)
ECG: electrocardiography
ESR: erythrocyte sedimentation rate
INR: international normalized ratio
ISARIC: International Severe Acute Respiratory and Emerging Infection Consortium
LDH: lactate dehydrogenase
MIS-C: multisystem Inflammatory Syndrome in children
PIMS-TS: paediatric inflammatory multisystem syndrome temporally associated with COVID-19
PCR: Polymerase chain reaction
SARS-CoV-2: severe acute respiratory syndrome coronavirus2
