# Supplementary material for: Short-term and medium-term clinical outcomes of multisystem inflammatory syndrome in children: a prospective observational cohort study
Source: Ital J Pediatr. 2024 Jan 4;50:1. doi: 10.1186/s13052-023-01569-7 (PMC10768316; doi:10.1186/s13052-023-01569-7)
Supplement: Supplementary file 1 — Additional file 1: Supplementary, Appendix: Table A1. MIS-C/PIMS criteria; Table A2. Number of children receiving different treatment options during hospitalisation; Table A3. The number of children with abnormally high and low laboratory test results revealed during the hospital stay; Table A4. Laboratory parameters across timepoints. Acute phase refers to the worst laboratory results during hospitalisation; Table A5. Findings on chest CT and echocardiography; Table A6. Frequency of symptoms across timepoints; Box A1. Definitions of echocardiographic changes; Fig. A1. Box plots and parallel plots representing changes in serum markers over time: during the acute MIS-C phase and at ‘Follow-up 1’ (2 weeks after discharge) and ‘Follow-up 2’ (6 weeks after discharge). [file 13052_2023_1569_MOESM1_ESM.docx]

**Appendix A**

The appendix is an optional section that can contain details and data supplemental to the main text—for example, explanations of experimental details that would disrupt the flow of the main text but nonetheless remain crucial to understanding and reproducing the research shown; figures of replicates for experiments of which representative data is shown in the main text can be added here if brief, or as Supplementary data. Mathematical proofs of results not central to the paper can be added as an appendix.

Table A1. MIS-C/PIMS criteria

|  | **ISARIC/WHO** | **US, CDC** | **UK, RCPCH** |  |
| --- | --- | --- | --- | --- |
| **Age** | 0-19 | <21 |  |  |
| **Fever** | ≥3 days | >=38 (including subjective) | Persistent fever >38.5°C |  |
|  | AND | | |  |
| **Laboratory evidence of inflammation** | ESR, C-reactive protein or procalcitonin | Elevated C-reactive protein | neutrophilia, elevated CRP and lymphopenia |  |
|  | AND two or more of the following: | AND |  |  |
| **Multisystem organ involvement** | Rash OR bilateral non-purulent conjunctivitis OR muco-cutaneous inflammation signs (oral, hands and feet) | multisystem (>=2) organ involvement (cardiac, hematologic, gastrointestinal, mucocutaneousor shock) | single or multi-organ dysfunction (shock, cardiac,  respiratory, renal, gastrointestinal or neurological disorder) with additional features |  |
|  | Hypotension or shock |  |  |  |
|  |  |  |  |  |
|  | Features of myocardial dysfunction, or pericarditis, or valvulitis, or coronary abnormalities (clinical features, ECHO findings, or laboratory markers such as elevated Troponin/NT-proBNP) |  |  |  |
|  |  |  |  |  |
|  | Acute gastrointestinal problems (such as diarrhoea, vomiting or abdominal pain) |  |  |  |
|  | Evidence of coagulopathy (such as abnormal PT, PTT, elevated d-Dimers) |  |  |  |
|  | AND | AND  One of the following: |  |  |
| **Evidence of current or previous COVID-19** | (RT-PCR, antigen test or serology positive) or likely contact with patients with COVID | 1. SARS-CoV-2 positive RT-PCR test  2. SARS-CoV-2 positive antibody test  3. SARS-CoV-2 positive antigen test  4. Close contact with a confirmed or probable case of COVID-19 disease | SARS-CoV-2 PCR testing may be positive or negative |  |
|  | AND | | |  |
| **No other obvious microbial cause of inflammation** | including bacterial sepsis, staphylococcal or streptococcal shock syndromes |  | including bacterial sepsis, staphylococcal or streptococcal shock syndromes, infections associated with myocarditis such as enterovirus |  |
| **Link** | <https://www.who.int/news-room/commentaries/detail/multisystem-inflammatory-syndrome-in-children-and-adolescents-with-covid-19> | <https://www>.cdc.gov/mis/mis-c/hcp_cstecdc/index.html | <https://www.rcpch.ac.uk/resources/paediatric-multisystem-inflammatory-syndrome-temporally-associated-covid-19-pims-guidance> |  |

# Table A2. Number of children receiving different treatment options during hospitalisation.

| Treatment | Number of children |
| --- | --- |
| Antibiotics | 37 (100%) |
| Anticoagulants | 34 (91.9%) |
| Corticosteroids | 33 (89.2%) |
| Intravenous fluids | 33 (89.2%) |
| IVIG | 32 (86.5%) |
| Antivirals | 2 (5.4%) |
| Oxygen supplementation therapy | 10 (27%) |
| Inotropes/vasopressors | 14 (37.8%) |
| High dependency unit admission | 18 (48.6%) |
| The number of days spent in the high dependency unit (median, IQR) | 5.5 (4-7) |
| Blood transfusions | 8 (21.6%) |
| Invasive ventilation | 3 (8.1%) |
| Renal replacement/dialysis | 2 (5.4%) |

# Table A3. The number of children with abnormally high and low laboratory test results revealed during the hospital stay.

| Parameter | MIS-C hospitalisation | Follow-up 1  15 (14-18) days post-discharge  n=33 | Follow-up 2 47 (41-52) days post-discharge  n=25 |
| --- | --- | --- | --- |
| Inflammatory markers |  |  |  |
| CRP ** | 37 (100%) | 0/32 (0%) | 1/25 (4%) |
| ESR ** | 33 (89.2%) | 14/31 (45.2%) | 4/24 (16.7%) |
| D-dimer ** | 35 (94.6%) | 2/23 (8.7%) | 0/18 (0%) |
| Ferritin ** | 31 (83.8%) | 6/27 (22.2%) | 2/22 (9.1%) |
| LDH level ** | 18 (48.6%) | 5/32 (15.6%) | 0/24 (0%) |
| Whole blood cell count |  |  |  |
| Haemoglobin * | 33 (89.2%) | 6 (18.2%) | 3 (12%) |
| Hematocrit * | 34 (91.9%) | 23 (69.7%) | 14 (56%) |
| WBC ** | 27 (73%) | 0 (0%) | 0 (0%) |
| Platelets ** | 34 (91.9%) | 15 (45.5%) | 20 (80%) |
| Platelets * | 29 (78.4%) | 2 (6.1%) | 0 (0%) |
| Neutrophils ** | 35 (94.6%) | 1 (3%) | 1 (4 %) |
| Lymphocytes * | 23 (62.2%) | 0 (0%) | 0 (0%) |
| Coagulation |  |  |  |
| aPTT ** | 17 (45.9%) | 2/27 (7.4%) | 1/19 (5.3%) |
| PT ** | 28 (75.7%) | 2/27 (7.4%) | 0/19 (0%) |
| INR ** | 23 (62.2%) | 0/27 (0%) | 0/19 (0%) |
| Fibrinogen ** | 28 (75.7%) | 2/27 (7.4%) | 0/18 (0%) |
| Biochemical profile |  |  |  |
| Creatinine ** | 6/37 (16.2%) | 1/26 (3.8%) | 0/19 (0%) |
| Albumin * | 34 (91.9%) | 0/32 (0%) | 0 (0%) |
| Urea ** | 16 (43.2%) | 3/32 (9.4%) | 0 (0%) |
| AST level ** | 24 (64.9%) | 2/32 (6.3%) | 0 (0%) |
| ALT ** | 28 (75.7%) | 4/31 (12.9%) | 0 (0%) |
| Troponin ** | 23/31 (74.2%) | 3/15 (20%) | 0/15 (0%) |
| Creatine Kinase * | 6/36 (16.7%) | 0/26 (0%) | 1/18 (5.6%) |

* the lowest level of the parameter during all hospital stay of each patient; ** the highest level of the parameter during all hospital stay of each patient.

WBC, white blood cells; APTT, Activated Partial thromboplastin time; PTT, Prothrombin Time; INR, International normalized ratio; CRP, C-reactive protein; ESR, Erythrocyte sedimentation rate; LDH, Lactate dehydrogenase; ALT, Alanine aminotransferase; AST, Aspartate aminotransferase.

Table A4. Laboratory parameters across timepoints. Acute phase refers to the worst laboratory results during hospitalisation. All values are presented as median (interquartile range).

| Parameter | MIS-C hospitalisation (n=37) | Follow-up 1  15 (14-18) days post-discharge  (n=33) | Follow-up 2  47 (41-52) days post-discharge  (n=25) |
| --- | --- | --- | --- |
| Inflammatory markers | | | |
| CRP** (mg/L) | 195.3 (120.0-296.4) | 0.5 (0.2-1.0) (n=32) | 0.5 (0.2-0.8) |
| ESR** (mm/h) | 59.0 (30.0-76.0) | 21.0 (8.5-27.0) (n=31) | 9.0 (3.0-12.8) (n=24) |
| D-dimer** (mg/L) | 1.2 (1.0-2.0) | 0.1 (0.0-0.2) (n=23) | 0.1 (0.0-0.1) (n=18) |
| Ferritin** (ug/L) | 340.0 (277.7-734.4) | 43.1 (20.6-84.4) (n=27) | 12.7 (9.3-29.6) (n=22) |
| LDH** (U/L) | 317.0 (268.0-373.0) | 223.5 (206.2-264.0) (n=32) | 225.5 (198.8-241.5) (n=24) |
| Whole blood cell count |  |  |  |
| Haemoglobin* (g/L) | 94.0 (84.0-102.0) | 116.0 (110.0-127.0) | 124.0 (116.0-128.0) |
| Haematocrit* (%) | 28.7 (26.3-30.2) | 35.2 (34.0-38.0) | 37.6 (35.2-39.7) |
| WBC** (10^9/L) | 18.3 (13.7-22.6) | 6.0 (4.8-7.9) | 6.6 (5.3-7.7) |
| Platelets** (10^9/L) | 587.0 (504.0-698.0) | 314.0 (266.0-466.0) | 364.0 (328.0-410.0) |
| Platelets* (10^9/L) | 112.0 (76.0-160.0) |  |  |
| Neutrophils** (10^9/L) | 11.6 (7.9-16.2) | 2.3 (1.8-3.1) | 2.8 (2.0-3.9) |
| Lymphocytes* (10^9/L) | 0.9 (0.7-1.4) | 2.5 (1.9-3.2) | 2.6 (1.81-3.1) |
| Coagulation |  |  |  |
| APTT** (sec) | 37.1 (31.6-46.8) | 31 (28.7-33.3) (n=27) | 31.9 (30.4-34.0) (n=19) |
| PT**(sec) | 15.0 (14.0-17.0) | 12.0 (11.4-12.4) (n=27) | 12.5 (12.0-12.6) (n=19) |
| INR** | 1.3 (1.2-1.5) | 1.0 (1.0-1.1) (n=27) | 1.1 (1.0-1.1) (n=19) |
| Fibrinogen **(g/L) | 5.0 (4.4-6.4) | 2.9 (2.7-3.2) (n=27) | 2.7 (2.5-3.2) (n=18) |
| Biochemical profile |  |  |  |
|  |  |  |  |
| Creatinine** (umol/L) | 59.0 (46.0-68.7) | 47.0 (41.5-57.8) (n=26) | 47.0 (41.0-59.0) (n=19) |
| Albumin* (g/L) | 24.5 (21.0-27.0) | 41.2 (39.9-42.0) (n=32) | 41.8 (40.0-43.3) |
| Urea**(mmol/L) | 7.0 (5.5-9.3) | 4.2 (3.2-5.2) (n=32) | 4.2 (3.3-4.8) |
| Sodium** (mmol/L) | 142.0 (140.0-144.0) | 140.0 (138.2-141.0) (n=30) | 140.0 (138.0-142.0) |
| Potassium**(mmol/L) | 4.9 (4.5-5.4) | 4.5 (4.2-4.8) (n=29) | 4.3 (4.1-4.6) |
| Total bilirubin** (umol/L) | 9.6 (7.1-17.9) (n=36) | 7.6 (5.8-10.6) (n=29) | 6.2 (5.1-10.0) (n=24) |
| AST** (U/L) | 55.9 (39.9-96.0) | 31.3 (23.9-39.3) (n=32) | 27.4 (20.5-33.4) |
| ALT ** (U/L) | 81.6 (42.0-97.4) | 20.9 (15.75-24.4) (n=31) | 14.2 (11.7-16.4) |
| Troponin** (pg/ml) | 40.0 (11.2-84.0) (n=31) | 1.7 (0.0-5.2) (n=15) | 0.5 (0.1-1.0) (n=15) |
| Creatine kinase** (U/L) | 46.5 (29.5-108.5) (n=36) | 62.0 (43.2-100.5) (n=26) | 82.5 (71.0-123.0) (n=18) |

* the lowest level of the parameter during all hospital stay of each patient; ** the highest level of the parameter during all hospital stay of each patient.

WBC, white blood cells; APTT, Activated Partial thromboplastin time; PTT, Prothrombin Time; INR, International normalized ratio; CRP, C-reactive protein; ESR, Erythrocyte sedimentation rate; LDH, Lactate dehydrogenase; ALT, Alanine aminotransferase; AST, Aspartate aminotransferase.

Table A5. Findings on chest CT and echocardiography

| Finding | MIS-C hospitalisation | Follow-up 1  15 (14-18) days post-discharge | Follow-up 2 47 (41-52) days post-discharge |
| --- | --- | --- | --- |
| Infiltrates at chest CT scan | 9/26 (34.6%) | 0 (0%) | 0 (0%) |
| Myocardial dysfunction | 6 (16.2%) | 0/32 (0%) | 0/19 (0%) |
| Features of pericarditis | 5 (13.5%) | 0/32 (0%) | 0/19 (0%) |
| Coronary abnormalities | 2 (5.4%) | 2/32 (6.25%) | 1/19 (5.26%) |
| Features of valvulitis | 0 (0%) | 0/32 (0%) | 0/19 (0%) |

Definitions: Myocardial dysfunction, enlarged ventricular cavity AND reduced ejection fraction; Pericarditis, pericardial effusion over 5 mm; Coronary abnormalities, doctor’s diagnosed coronary artery dilation OR aneurisms; valvulitis, thickening/distortion of cusps AND presence of pathological effusion.

# Table A6. Frequency of symptoms across timepoints

| Symptom | MIS-C hospitalisation | Follow-up 1  15 (14-18) days post-discharge  n=33 | Follow-up 2 47 (41-52) days post-discharge  n=36 |
| --- | --- | --- | --- |
| Fever | 37 (100%) | 0 (0%) | 0 (0%) |
| Fatigue | 32 (86.5%) | 5 (15.2%) | 1 (2.8%) |
| Oral inflammation | 31 (83.8%) | 1 (3.0%) | 0/25 (0%) |
| Rash | 26 (70.3%) | 2 (6.1%) | 1 (2.8%) |
| Maculopapular type of rash | 14/26 (53.8%) | 1/2 (50%) | 0/1 (0%) |
| Scleritis | 23 (62.2%) | 0 (0%) | 0/25 (0%) |
| Tachycardia (according to the age) | 19 (51.4%) | 0 (0%) | 0/25 (0%) |
| Vomiting | 19 (51.4%) | 0 (0%) | 0 (0%) |
| Bilateral conjunctivitis | 16 (43.2%) | 0 (0%) | 0 (0%) |
| Cervical lymphadenopathy | 16 (43.2%) | 0 (0%) | 0/25 (0%) |
| Abdominal pain | 14 (37.8%) | 0 (0%) | 0 (0%) |
| Bilateral non-purulent conjunctivitis | 14 (37.8%) | 0 (0%) | 0 (0%) |
| Peripheral cutaneous inflammation signs | 14 (37.8%) | 0 (0%) | 0 (0%) |
| Tachypnoea | 13 (35.1%) | 1 (3.0%) | 0/25 (0%) |
| Irritability | 11 (29.7%) | 0 (0%) | 0 (0%) |
| Diarrhoea | 11 (29.7%) | 0 (0%) | 0 (0%) |
| Urinary problems | 10 (27%) | 0 (0%) | 0 (0%) |
| Stiff neck | 10 (27%) | 0 (0%) | 0 (0%) |
| Sore throat | 8 (21.6%) | 0 (0%) | 0 (0%) |
| Pale/mottled skin | 8 (21.6%) | 0 (0%) | 0 (0%) |
| Prolonged capillary refill time (>2 seconds) | 8 (21.6%) | 0 (0%) | 0/25 (0%) |
| Urinary output < 2 mL/kg/hr | 7 (18.9%) | 0 (0%) | 0/25 (0%) |
| Hypotension (according to the age) | 7 (18.9%) | 0 (0%) | 0 (0%) |
| Cough | 6 (16.2%) | 0 (0%) | 0 (0%) |
| Hypotonia/floppiness | 6 (16.2%) | 0 (0%) | 0 (0%) |
| Headache | 6 (16.6%) | 1 (3.0%) | 0 (0%) |
| Runny nose | 5 (13.5%) | 0 (0%) | 0 (0%) |
| Cold hands/feet | 4 (10.8%) | 0 (0%) | 0 (0%) |
| Respiratory distress | 4 (10.8%) | 0 (0%) | 0 (0%) |
| Muscle aches | 4 (10.8%) | 0 (0%) | 0 (0%) |
| Chest pain | 2 (5.4%) | 0 (0%) | 0 (0%) |
| Wheezes in the lungs | 2 (5.4%) | 0 (0%) | 0/25 (0%) |
| Photophobia | 2 (5.4%) | 0 (0%) | 0 (0%) |
| Unable to drink | 1 (2.7%) | 0 (0%) | 0 (0%) |
| Swollen joints | 1 (2.7%) | 0 (0%) | 0 (0%) |
| Joint pain | 0 (0%) | 1 (3.0%) | 0 (0%) |
| Skin ulcers | 0 (0%) | 0 (0%) | 0 (0%) |
| Seizures | 0 (0%) | 0 (0%) | 0 (0%) |
| Paralysis | 0 (0%) | 0 (0%) | 0/25 (0%) |
| Hyposmia/anosmia | 0 (0%) | 0 (0%) | 0 (0%) |
| Hypogeusia | 0 (0%) | 0 (0%) | 0 (0%) |
| Bleeding (haemorrhage) | 0 (0%) | 0 (0%) | 0 (0%) |
| Number of children with 0 symptom | 0 (0%) | 26 (78.8%) | 34 (94.4%) |
| Number of children with 1 symptom | 0 (0%) | 3 (9.1%) | 2 (5.6 %) |
| Number of children with 2 symptoms | 0 (0%) | 4 (12.1%) | 0 (0%) |
| Number of children with ≥3 symptoms | 37 (100%) | 0 (0%) | 0 (0%) |

# Box A1. Definitions of echocardiographic changes

| Echocardiography findings | Definition |
| --- | --- |
| Features of myocardial dysfunction | Enlarged ventricular cavity AND reduced ejection fraction |
| Features of pericarditis | Pericardial effusion over 5 mm |
| Features of valvulitis | Thickening/distortion of cusps AND presence of pathological effusion |
| Coronary abnormalities | Doctor’s diagnosed coronary artery dilation OR aneurisms |

Figure A1. Box plots and parallel plots representing changes in serum markers over time: during the acute MIS-C phase and at ‘Follow-up 1’ (2 weeks after discharge) and ‘Follow-up 2’ (6 weeks after discharge).
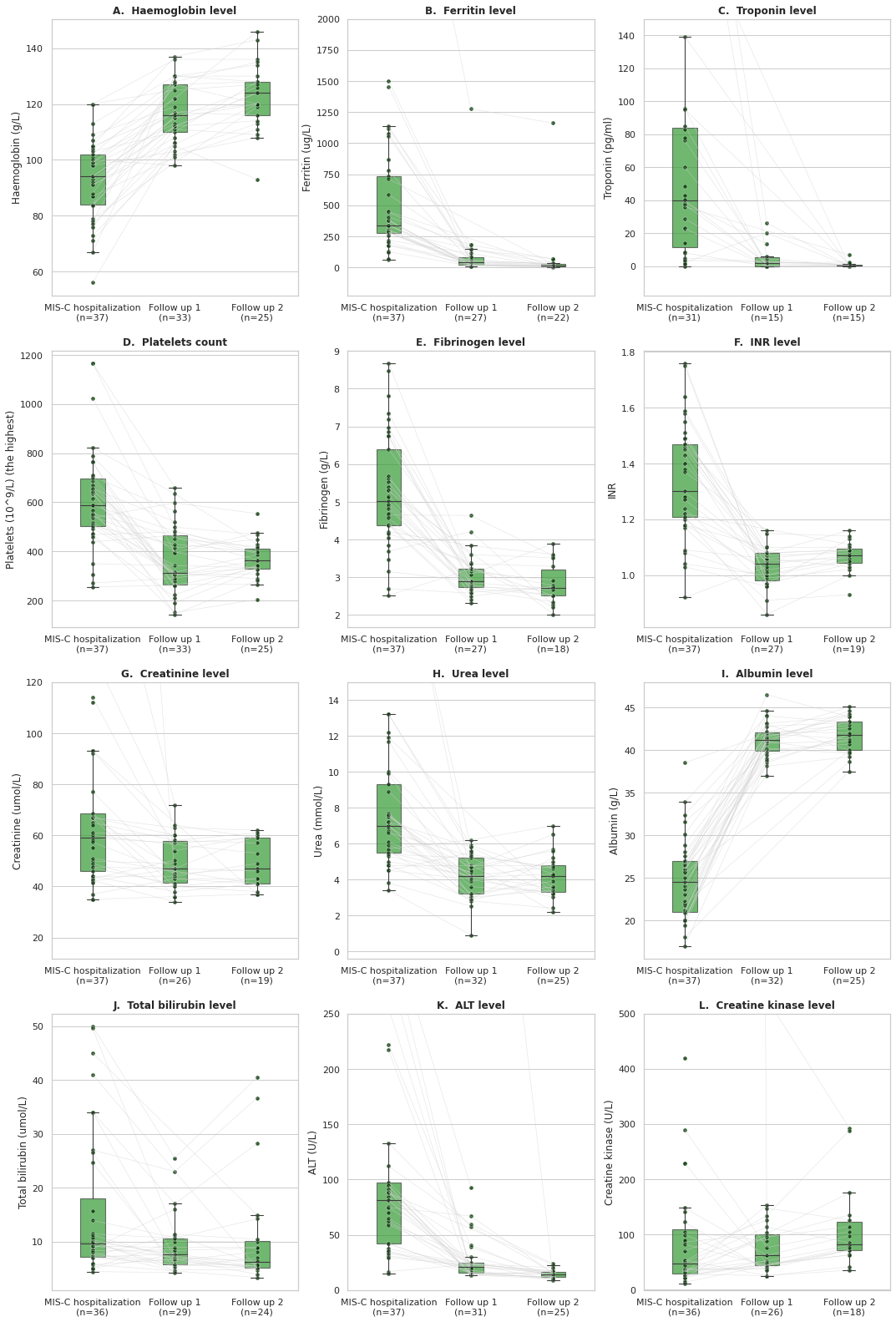


*The horizontal lines in the boxes indicate medians; lower and upper edges of boxes indicate interquartile range and the bars extend to the highest and lowest value within 1.5 times the interquartile ranges.*
